# Dexamethasone Attenuates Hyperexcitability Provoked by Experimental Febrile Status Epilepticus

**DOI:** 10.1523/ENEURO.0430-19.2019

**Published:** 2019-11-15

**Authors:** Megan M. Garcia-Curran, Alicia M. Hall, Katelin P. Patterson, Manlin Shao, Nihal Eltom, Kevin Chen, Celine M. Dubé, Tallie Z. Baram

**Affiliations:** 1 Department of Anatomy and Neurobiology, University of California-Irvine, Irvine, California 92697; 2 Department of Pediatrics, University of California-Irvine, Irvine, California 92697; 3 Department of Neurology, University of California-Irvine, Irvine, California 92697

**Keywords:** blood–brain barrier, cytokines, epileptogenesis, febrile seizures, neuroinflammation, translational

## Abstract

The role of neuroinflammation in the mechanisms of epilepsy development is important because inflammatory mediators provide tractable targets for intervention. Inflammation is intrinsically involved in the generation of childhood febrile seizures (FSs), and prolonged FS [febrile status epilepticus (FSE)] precedes a large proportion of adult cases of temporal lobe epilepsy (TLE). As TLE is often refractory to therapy and is associated with serious cognitive and emotional problems, we investigated whether its development can be prevented using anti-inflammatory strategies. Using an immature rat model of FSE [experimental FSE (eFSE)], we administered dexamethasone (DEX), a broad anti-inflammatory agent, over 3 d following eFSE. We assessed eFSE-provoked hippocampal network hyperexcitability by quantifying the presence, frequency, and duration of hippocampal spike series, as these precede and herald the development of TLE-like epilepsy. We tested whether eFSE provoked hippocampal microgliosis, astrocytosis, and proinflammatory cytokine production in male and female rats and investigated blood–brain barrier (BBB) breaches as a potential contributor. We then evaluated whether DEX attenuated these eFSE sequelae. Spike series were not observed in control rats given vehicle or DEX, but occurred in 41.6% of eFSE-vehicle rats, associated with BBB leakage and elevated hippocampal cytokines. eFSE did not induce astrocytosis or microgliosis but provoked BBB disruption in 60% of animals. DEX significantly reduced spike series prevalence (to 7.6%) and frequency, and abrogated eFSE-induced cytokine production and BBB leakage (to 20%). These findings suggest that a short, postinsult intervention with a clinically available anti-inflammatory agent potently attenuates epilepsy-predicting hippocampal hyperexcitability, potentially by minimizing BBB disruption and related neuroinflammation.

## Significance Statement

Epilepsy is the third most common brain disorder. Temporal lobe epilepsy (TLE) involves limbic circuits and commonly provokes serious cognitive and emotional problems. Because TLE is often refractory to therapy, its prevention would be a powerful and clinically important strategy. A large proportion of TLE follows long childhood febrile seizures [febrile status epilepticus (FSE)], which intrinsically involves neuroinflammation. Here we used dexamethasone (DEX), a broad anti-inflammatory agent, commencing after experimental FSE (eFSE). Transient DEX administration significantly reduced the prevalence and frequency of spike-series, a surrogate marker of network hyperexcitability. The mechanism likely involved the repression by DEX of eFSE-induced cytokine production and blood–brain barrier disruption. Our data demonstrate the efficacy of mechanism-driven post-insult intervention in mitigating epileptogenesis, with significant translational potential

## Introduction

Temporal lobe epilepsy (TLE) is the most common human focal epilepsy. TLE is characterized by hippocampal seizures and is associated with cognitive and emotional problems. Prolonged childhood febrile seizures [febrile status epilepticus (FSE)] precede a majority of adult TLE (Cendes et al., 1993; French et al., 1993) and may contribute to TLE development (Annegers et al., 1987; Cendes et al., 1993; Dubé et al., 2007; Lewis et al., 2014; Yokoi et al., 2019). To elucidate potential mechanisms by which FSE promotes epilepsy, immature rat models of experimental FSE (eFSE) have been created (Baram et al., 1997; Heida et al., 2004; Heida and Pittman, 2005; Dubé et al., 2010b; Reid et al., 2013). The FSE model developed by the authors promotes epilepsy in ∼40% of rats, similar to findings in humans (Dubé et al., 2006, 2010b; Choy et al., 2014).

Inflammation has been implicated in both the development of epilepsy (epileptogenesis) and its progression and comorbidities (Vezzani et al., 2011b). Inflammatory markers have been identified in hippocampi removed from individuals with TLE and in animal models (Aronica and Gorter, 2007; Dubé et al., 2007; Balosso et al., 2013). Inflammation is intrinsically involved in FSE because of its role in fever generation (Luheshi et al., 1997; Cartmell et al., 1999; Reid et al., 2009; Eskilsson et al., 2014).

Previous work has demonstrated that the cytokine interleukin (IL)-1β is a crucial component of eFSE generation (Dubé et al., 2005; Heida and Pittman, 2005). In addition, higher hippocampal IL-1β levels were found in animals that become epileptic after eFSE (Dubé et al., 2010b). Notably, rats destined to become epileptic had increased hippocampal levels of several proinflammatory molecules within hours following eFSE (Patterson et al., 2015). These included cyclooxygenase 2 (COX2), GFAP (an astrocyte activation marker), TNF-α, and IL-1 receptor 1 (IL-1R1; Dubé et al., 2005, 2007, 2010b; Patterson et al., 2015). Thus, inflammation is an attractive target for antiepileptogenic therapies aiming to abort the process initiated by FSE and prevent spontaneous seizures (Vezzani et al., 2011b; Dedeurwaerdere et al., 2012; Jiang et al., 2013; Patterson et al., 2014).

Neuroinflammation often provokes blood–brain barrier (BBB) breaches because inflammatory cytokines, prostaglandins and other mediators disrupt endothelial tight junctions that contribute to the BBB (Abbott, 2000). Increased BBB permeability allows the entry of molecules and cells from peripheral tissues. These further enhance inflammation and the risk of future seizures (Seiffert et al., 2004; Friedman, 2011; Janigro, 2012), likely because the peripheral cells and molecules promote neuronal injury and further inflammation, creating a vicious injurious cycle (Gorter et al., 2019). Importantly, several insults that promote epileptogenesis lead to early BBB breaches (Tomkins et al., 2008; Bar-Klein et al., 2017). Yet, whether eFSE causes breakdown of the BBB and whether anti-inflammatory agents mitigate these potential BBB disruptions has not been studied.

Several interrelated inflammatory cascades are triggered by FSE (Vezzani et al., 1999; Reid et al., 2009; Patterson et al., 2015; Brennan et al., 2016). The interactions among these pathways are complex, including feedback and compensation (Vezzani et al., 2011b). Therefore, it is not surprising that targeting specific pathways has had little efficacy in preventing eFSE-induced hyperexcitability (Brennan et al., 2016; Curran et al., 2016), though success has been reported in attenuating epileptogenesis in adults (Jung et al., 2006; Maroso et al., 2010; Vezzani et al., 2011a). Here, we administered the global anti-inflammatory agent dexamethasone (DEX) shortly after eFSE for a limited period, testing whether it suppressed inflammatory cytokines and the formation of hyperexcitable networks (Dubé et al., 2000). DEX, a synthetic glucocorticoid, inhibits expression of many cytokine genes, including IL-1β (Jeon et al., 2000; Jang et al., 2007), via the glucocorticoid receptor (GR; Reul et al., 1987). While varied downstream effects of GR activation occur (Datson et al., 2008; Groeneweg et al., 2012), the inhibition of nuclear factor-κB and a consequent transcription block of key proinflammatory molecules likely mediate the anti-inflammatory effects of DEX (Auphan et al., 1995; Gilmore, 2006). We found that transient DEX administration after eFSE suppressed hippocampal hyperexcitability, likely by preventing BBB leakage and elevation of hippocampal cytokines.

## Materials and Methods

### Experimental overview

The goal of these experiments was to examine the role of inflammatory processes, including BBB breaches, in the proepileptogenic cascade that follows eFSE and culminates in spontaneous seizures. Practical feasibility issues governed the design of our experiments. Specifically, only 40% of eFSE-experiencing rats develop spontaneous seizures, and these are sparse, averaging less than one seizure per week (Choy et al., 2014). Thus, it was prohibitively costly in labor, time, and expense to perform an adequately powered study that aimed to demonstrate significant reduction in seizure frequency from an already low frequency, or reduction in the proportion of rats that develop epilepsy when that proportion is only 33–40%. Specifically, power analysis showed that to detect a 50% reduction from a 40% seizure probability with an 0.8 power and α = 0.05 would require group sizes of 199 rats (G*Power, version 3.1.9.4).

Therefore, we used a surrogate marker approach that is typical and acceptable in such scenarios: we identified a marker of epileptogenesis, namely spike series, and examined the effects of DEX on this epilepsy-predicting marker.

Four experiments were conducted. Experiment 1 determined the relation of spike series and spontaneous seizures following eFSE. eFSE-experiencing rats and control littermates were implanted with bilateral hippocampal electrodes 2 months after the eFSE. Starting at approximately postnatal day 100 (P100), rats were chronically recorded using video/EEG to detect spike series and seizures. Rats were recorded in this manner until they were ∼1 year of age.

Experiment 2 was performed after the first experiment established that spike series always preceded spontaneous seizures provoked by eFSE. This experiment assessed the effects of DEX on spike series, a measure of hippocampal hyperexcitability after eFSE. The cohort was implanted with EEG electrodes within 3 d after eFSE, and video/EEGs were conducted for 4 weeks. Experimental groups consisted of: control (non-eFSE) rats given vehicle (CTL-VEH); DEX-treated control (non-eFSE) rats (CTL-DEX); eFSE rats given vehicle (eFSE-VEH); and eFSE rats provided with DEX (eFSE-DEX).

Experiment 3 examined the effects of *post hoc* DEX on the acute proinflammatory cascades initiated by eFSE. Controls and eFSE rats were included, and subgroups of both received DEX immediately after the eFSE. Rats were killed 3 h after the end of eFSE and hippocampi were processed to measure mRNA levels of several proinflammatory mediators using RT-PCR.

Experiment 4 examined whether eFSE leads to the breakdown of the BBB, and whether DEX administration after the eFSE might attenuate BBB disruption. Rats (*n* = 25) were randomly assigned to undergo eFSE on P11 or to serve as controls. Rats of both groups received either vehicle or DEX following the eFSE. Twenty-four hours later, rats underwent perfusion with fluorescein isothiocyanate (FITC)-conjugated albumin (FITC-albumin) and then killed to examine for evidence of BBB leakage.

### Procedure of experimental febrile status epilepticus

All animal procedures were approved by the University of California-Irvine animal care committee and performed according to NIH guidelines. The induction of eFSE has been described in detail previously (Baram et al., 1997; Dubé et al., 2010b; Patterson et al., 2015). Briefly, Sprague Dawley rat pups were used for all studies on postnatal day 10 or 11 based on their weight (Harlan; RRID:RGD_5508397). Pups were placed, two at a time, inside a 3 L glass container lined with absorbent paper. Pups were subjected to a continuous stream of warm air until behaviors indicating seizures began. These behaviors were a sudden arrest of hyperthermia-induced hyperkinesis (freezing) followed by chewing automatisms. Core temperatures at the onset of the seizures (typically, ∼38.5°C) were rapidly measured and seizure-onset time was noted. Once seizures commenced, elevated core temperature and seizures were maintained via the warm air stream for 40–60 min. Seizure behaviors typically progressed over the hyperthermia period, including mild chewing, clonic movements, and eventual tonic extension. Core temperature was measured every 2 min during the eFSE. If the core temperature of the pups exceeded 41.5°C, they were removed from the chamber and placed on a cool metal surface for the next 2 min. Cessation of the hyperthermia promptly led to termination of the eFSE and was facilitated by briefly immersing pups in cool water (∼23.0°C). Pups were then dried and placed on a euthermic pad maintained at 37°C for 20 min then returned to their home cage and dam. In this study, all pups subjected to hyperthermia experienced eFSE and there was no eFSE-related mortality.

### DEX administration

Following the eFSE and cooling, rats were administered an intraperitoneal injection of DEX (3 mg/kg; Sigma-Aldrich). The DEX dose was based on a large body of literature using a wide range of doses of DEX for multiple purposes (0.1–10 mg/kg). We chose 3 mg/kg because it had been shown to fully suppress inflammation, and used a short, tapered treatment course to avoid many of the side effects (Quan et al., 2000; Duffy et al., 2014; Tsai et al., 2016). Aldosterone was administered subcutaneously (0.2 μg/100 mg; Acros Organics) to provide mineralocorticoid functional support as DEX blocks endogenous corticosterone production (Brunson et al., 2001).

Rats undergoing video-EEGs (experiment 2) received subsequent tapering doses of DEX (together with aldosterone) over 48 h as follows: 1.5 mg/kg DEX 24 h after eFSE; 0.75 mg/kg DEX 48 h after eFSE. Aldosterone was continued for the next 4 d (seven treatments total) to provide mineralocorticoid support during the potential suppression of the adrenal glands of the rats. Rats used for cytokine assessments (experiment 3) received a single DEX dose and were killed 3 h after the end of eFSE. Vehicle controls consisted of saline administered intraperitoneally.

### EEG electrode implantation

For the first experiment, electrodes were implanted 2 months after eFSE. For the second experiment, electrodes were implanted 3 d after eFSE. Rats were anesthetized under 1–3% isoflurane (Piramal Health Care) and bipolar electrodes (Plastics One) were permanently inserted into both dorsal hippocampi [with reference to bregma: P14: anteroposterior (AP), 2.7 mm; lateral (L), 1.8 mm; ventral (V), 2.2 mm; adult: AP, −3.6 mm; L, 2.6 mm; V, −2.8 mm]. A cortical electrode was placed over the parietal cortex (P14: AP, 2.0 mm; L, −1.5 mm; adult AP: 2.5 mm; L, 2.0 mm) and an additional ground electrode was inserted over the cerebellar cortex. The whole cap was secured with OrthoJet Dental Cement (Lang Dental). Following surgery, rats were kept on a warm pad for 1 h to recover from anesthesia and then returned to their home cages.

### Video/EEG monitoring and analyses

All rats were recorded using video/EEG to examine for eFSE-provoked alterations in hippocampal brain-wave activity and the potential mitigating effects of DEX. Synchronized videos served to categorize seizures using the Racine scale, and, in some cases, to further exclude potential movement artifact (e.g., from grooming).

Electrophysiological data were obtained at a frequency band of 1–200 Hz and sampled at 400 Hz using Powerlab 8SP (AD Instruments; RRID:SCR_001620) equipped with Chart 7 for Windows. The EEG tracings were synchronized with video monitoring using a commercial video camera (ZR40 Camcorder, Canon).

Rats from the first experiment were chronically recorded from ∼100 d after eFSE until the rats were 1 year of age. The cohort has been described in the study by Dubé et al. (2010b); however, the analyses reported have not been published previously. For the second experiment, video-EEG recordings commenced 2 d after electrode implantation and continued for 4 weeks. Rats were recorded for 2 h/d in amply bedded cages. These short recording durations were necessary to ensure that the pups received sufficient nutrition and maintained normal growth curves. The order of recording of individual rats and experimental groups was rotated daily to mitigate the potential confounding effects of diurnal variation in hyperexcitability. Four rats were monitored at a time, allowing for concurrent recording of littermate controls and eFSE animals. Hippocampal EEGs were acquired from freely moving pups and were synchronized with video via both the internal computer timer and a second, synchronized, clock that was placed within the field of view of the video camera.

### EEG analyses

EEGs were acquired, registered, and analyzed using LabChart 7 software (AD Instruments; RRID:SCR_001620), and the records were re-evaluated using an in-house algorithm (MATLAB, MathWorks; RRID:nlx_153890; available on request). All video/EEGs were analyzed without knowledge of treatment group by two trained investigators. Spikes were defined as deflections from a stable background with amplitudes more than twice that of the background rhythms and durations of 20–70 ms. Spike series were defined as a minimum four spikes occurring at a regular or semiregular interval from a stable background within <5 s.

### FITC-albumin administration and analyses of blood–brain barrier integrity

FITC-albumin was used as a direct method to investigate BBB permeability (Krueger et al., 2015; Breuer et al., 2017). In preliminary experiments, we found that, on occasion, the BBB was not fully mature in P10 rats. Therefore, in this experiment, eFSE was always imposed on P11 and BBB was examined at P12. Briefly, 24 h after eFSE, rats (both male and female) underwent terminal anesthesia induced with pentobarbital (Euthasol, Patterson Scientific). FITC-albumin was diluted to 10 mg/ml in PBS, infused into the left ventricle at a rate of 1 ml/min (10 mL/kg), and allowed to circulate for 5 min. Rats were then rapidly decapitated, and brains were removed and postfixed overnight in 4% paraformaldehyde. Brains were cryoprotected in 30% sucrose, frozen, and sectioned coronally at 30 μm thickness.

### Assessment of BBB breaches

A series of coronal sections from the septum through the superior colliculus (with reference to bregma: anterior, ∼0.5 mm; posterior, 4.5 mm) was evaluated for evidence of fluorescence extravasation by two observers who were unaware of group assignment. Sections were viewed at 100× and suspicious areas were magnified. A BBB breach was defined as FITC-albumin extravasation (i.e., fluorescence that was visible outside of clear vascular boundaries) in areas free of tissue tears. The presence of fluorescence inside glia was examined as well, and autofluorescence was excluded using appropriate filters. The numbers of extravasation areas per rat and their locations were noted.

### Quantitative real-time PCR

Whole hippocampi of both male and female rats, obtained 3 h after the cessation of eFSE, were rapidly dissected on ice using RNase-free instruments. Tissue was immediately transferred to prechilled centrifuge tubes kept on dry ice. Samples were stored at −80°C until use. Total RNA was extracted from hippocampi using the mirVana RNA Isolation Kit (Ambion) according to the manufacturer instructions. Double-stranded cDNA was synthesized from total RNA using the Roche First Strand cDNA Synthesis Kit (catalog #04379012001), with random hexamer primers. Analysis of the PCR products used cDNA samples in triplicate on a Roche Lightcycler 96 system. Samples were normalized to β-actin and quantified using the cycle threshold method (2^ΔΔCt^; Schmittgen and Livak, 2008). The primers used in this study are shown in Table 1.

**Table 1:** Primer designs

β-Actin	CAGCCTTCCTTCCTGGGTATGGAATC
	CAGCACTGTGTTGGCATAGAGGTCTTTAC
COX2	TGGTGCCGGGTCTGATGATG
	GCAATGCGGTTCTGATACTG
IL-1β	GTGAAATAGCAGCTTTCGACAGTGAGGAG
	GTGAGATTTGAAGCTGGATGCTCTCATCTG
IL-1R1	GCTTGTGACATCTTCGGCTA
	AATGAACCCAAGTAGCACTTTCA

### Immunohistochemistry

Rats were perfused under deep anesthesia as described above for the BBB experiments. Alternate serial sections from the same P12 rat brains used for FITC-albumin analyses were used to visualize microglia and astrocyte proliferation, which are cellular markers of neuroinflammation (Choy et al., 2014; Patterson et al., 2015). First, sections were quenched with 0.9% hydrogen peroxide for 30 min in 0.01 m PBS with 0.3% Triton X-100 (PBS-T) and blocked in 5% normal goat serum (NGS; catalog #R527-4001, Sigma-Aldrich) in 0.01 m PBS-T for 1 h. Then we incubated sections in primary antibody in 2% NGS in PBS-T overnight at 4°C followed by incubation with biotinylated mouse secondary antibody. Primary antibodies included mouse anti-GFAP (1:1000; Millipore) for delineating astrocytes and rabbit anti-P2Y12 (1:2000) for selective labeling of microglia. Secondary antibodies were goat anti-mouse (1:1000; catalog #BA-9200, Vector Laboratories; RRID:AB_2336171) for 1 h and goat anti-rabbit (1:400; catalog #BA-1000, Vector Laboratories; RRID:AB_2313606) for 1.5 h, respectively. Subsequently, incubation in Vectastain ABC-horseradish peroxidase kit (catalog #PK-6100, Vector Laboratories; RRID:AB_2336819) was performed for 2 h. We visualized the immunoreactive signals using a DAB (diaminobenzidine) Kit (catalog #SK-4100, Vector Laboratories; RRID:AB_2336382) following the manufacturer recommendations. Sections were mounted on slides and coverslipped using Permount Mounting Medium (catalog #SP15, Fisher Chemicals).

### Quantification of microglia and astrocytes

Quantification of microglia and astrocytes in hippocampal CA1 and CA3 was achieved using a 1200 × 1200 μm grid for CA1 and a standardized 16,400 μm^2^ trapezoid grid for CA3. The tops of the grids were centered on the “peak” of the CA1 pyramidal layer and on the most lateral point of area CA3b in coronal sections of the dorsal hippocampus. Counts and analyses of density were accomplished using the Fiji mode of ImageJ (NIH; RRID:SCR_002285). For microglia, P2Y12^+^ cells in the defined region were counted and compared among groups, and GFAP^+^ cells were quantified similarly for astrocytes. Analyses considered the effects of eFSE and of DEX.

### Statistical approaches and considerations

All analyses were performed without knowledge of treatment group. Statistical analyses were performed using Prism 7 Software (GraphPad; RRID:SCR_002798), and significance was set at <0.05. Datasets were initially analyzed for outliers using Grubb’s Outlier Test (α = 0.05), and outliers, found exclusively in the quantitative real-time PCR (qRT-PCR) data, were excluded. A two-way ANOVA with Tukey’s multiple-comparisons *post hoc* test was used to compare qRT-PCR results. Similarly, a two-way ANOVA was used to examine BBB leakage across eFSE and control rats with or without DEX treatment. To compare BBB breaches and the number of rats with and without spike series following eFSE, we used an on-line calculator for Barnard’s unconditional exact test because of its increased power for small sample sizes (Lydersen et al., 2009; Ahmed, 2013). The D’Agostino-Pearson Normality Test was used before ANOVA to ensure normality. The total number of spike series per animal and spike series frequency (per recorded hour) were compared using Kruskal–Wallis ANOVA for datasets without normal distribution. An observed versus expected goodness-of-fit analysis was used to compare the percentage of rats with spike series compared with previous short-term measures of brain changes following eFSE.

## Results

### Spike series, denoting a hyperexcitable network, precede spontaneous seizures after eFSE

**Figure 1.**
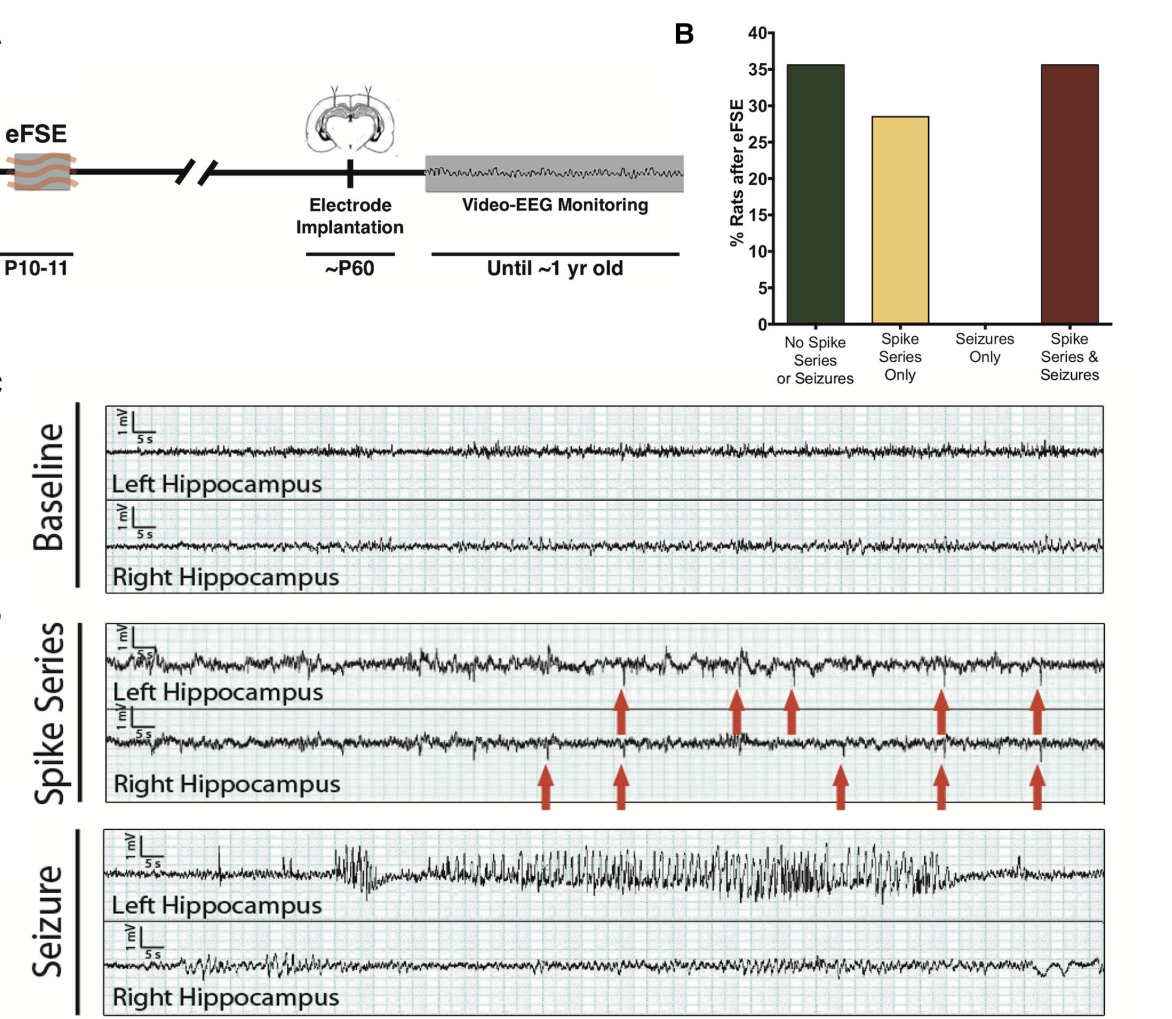
Spike series precede seizures in rats rendered epileptic by eFSE. ***A***, Experimental design. P10–P11 male rats underwent eFSE (*n* = 18) and electrode implantation at ∼P60. Rats were then recorded until 1 year of age. ***B***, eFSE-induced hyperexcitability in a proportion of rats, apparent as spike series alone (28.6% of rats that underwent eFSE; yellow bar) or spike series followed by seizures (35.7% of eFSE rats; red bar). No rat developed seizures before the onset of spike series. ***C–E***, Representative examples of baseline EEG of an adult rat that underwent eFSE, an example of a spike series, and an example of a seizure. Whereas the spike series and seizure emanate from the left hippocampus here and in Figure 2, there was no overall propensity to either side in the overall study and none in prior ones (Dubé et al., 2006, 2010b).

The current experiments aimed to determine whether the broad anti-inflammatory drug DEX prevented the epileptogenic process provoked by eFSE, which culminates in spontaneous seizures (epilepsy). However, only 40% of eFSE-experiencing rats develop spontaneous seizures. The spontaneous seizures are sparse (less than one seizure per week), and their onset is typically in young adult or adult rats, with a latency of >3 months (Dubé et al., 2006, 2010b; Choy et al., 2014), though seizures are rarely found earlier. Thus, the costs and duration of performing an appropriately powered study, looking at the reduction in seizure frequency from an already low level—or at a reduction in the proportion of rats that develop epilepsy when that proportion is only 33–40%—is prohibitive. Therefore, the initial experiments aimed to identify an earlier and more prevalent marker of the epileptogenic process. Specifically, we investigated whether spike series were generated by eFSE and whether spike series always preceded spontaneous seizures (Fig. 1*A*).

Spike series were never seen in our Sprague Dawley controls, as was apparent from over 2400 h of recording (Dubé et al., 2000, 2006, 2010b; Raol et al., 2003; Choy et al., 2014). Therefore, spike series can be used as a measure of abnormal hippocampal excitability in this context (Staley and Dudek, 2006; Staley et al., 2011). In the chronically video-EEG recorded eFSE-experiencing rats (*n* = 18), approximately a third had experienced neither recorded spike series nor seizures (30.8%). A total of 64.3% of rats developed spike series, and the majority of these (55.5%) progressed to spontaneous seizures (35.7% of all rats that experienced eFSE). Notably, no rat developed spontaneous seizures without prior spike series (Fig. 1*B*). Figure 1*C–E* provides representative EEGs from dorsal hippocampus of these eFSE rats. Figure 1*C* shows a typical multirhythm background. Figure 1*D* shows spike series that can be unilateral or propagate to both hippocampi. Figure 1*E* includes a typical hippocampal seizure involving the left hippocampus with little propagation, and we note that the rat was motionless during this ictal event. Together, the data in Figure 1 indicate that spike series precede spontaneous hippocampal seizures after eFSE and define an abnormal, hyperexcitable hippocampal network. Accordingly, spike series provide an early and useful target for assessing the efficacy of antiepileptogenic interventions.

### DEX attenuates the development and frequency of spike series after eFSE

**Figure 2.**
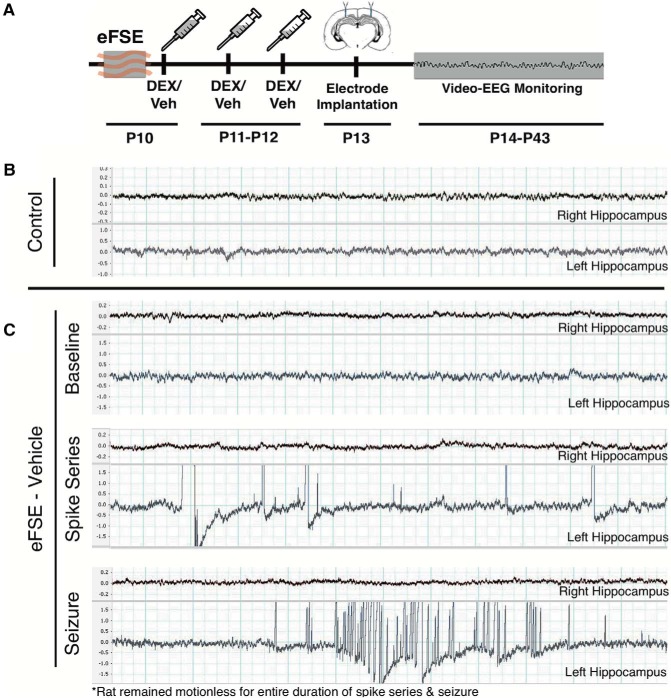
The effects of DEX on immature rat EEG following eFSE. ***A***, Experimental design: rats underwent eFSE at P10 or P11 and were then given tapering doses of DEX (1 h, 3 mg/kg; 24 h, 1.5 mg/kg; 48 h, 0.75 mg/kg). Mineralocorticoid support was provided by concurrent doses of aldosterone. Electrodes were implanted at P13, and rats were then video recorded with concurrent EEGs for 1 month. ***B***, Representative EEG from a control rat. ***C***, Representative traces of baseline EEG (top), a spike series (middle), and a single example of a seizure from a rat that had undergone eFSE and received vehicle treatment. For all EEG traces, the scale on left is in millivolts. Thin vertical bars delineate 0.25 s, and the bold vertical bars are at 1 s intervals.

**Figure 3.**
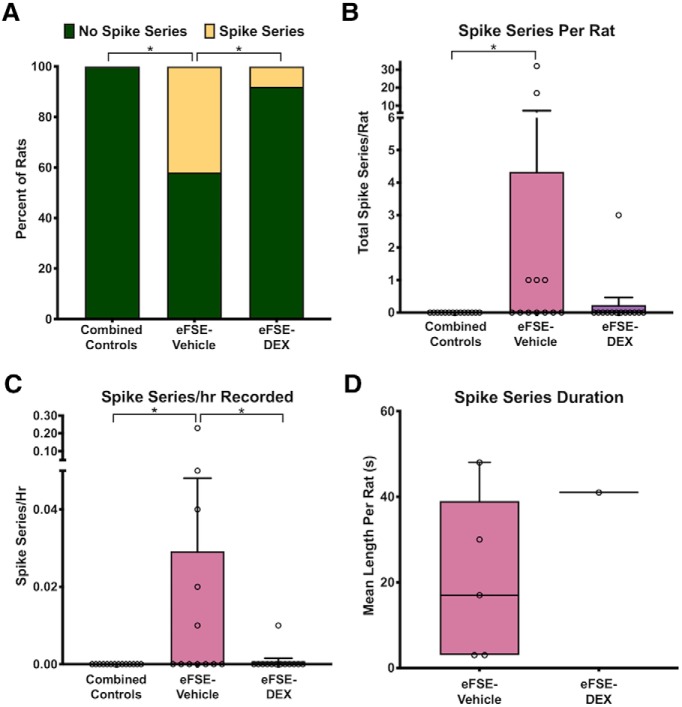
DEX administration following eFSE decreases hippocampal hyperexcitability. ***A***, DEX attenuated the proportion of rats with recorded spike series (unconditional Barnard’s exact test, control vs eFSE-VEH, *p* = 0.0062; eFSE-VEH vs eFSE-DEX, *p* = 0.031; control vs eFSE-DEX, *p* = 0.37). ***B***, DEX reduced the mean number of spike series per rat (Kruskal–Wallis ANOVA: mean rank difference, −8.083; *p* = 0.01; Dunn’s multiple-comparisons test, eFSE-DEX vs controls: mean rank difference, −1.53; *p* = 0.999; eFSE-VEH vs eFSE-DEX: mean rank difference, 6.55; *p* = 0.067). ***C***, DEX attenuated the mean spike series frequency (spike series/hour recorded) to control levels (CTL mean, 0 ± 0; eFSE-VEH mean, 0.029 ± 0.06; K-W ANOVA: K-W statistic, 9.71; mean rank difference, −8.29; *p* = 0.01; Dunn’s multiple-comparison test, eFSE-DEX vs eFSE-VEH: mean rank difference, 6.95; *p* = 0.046; eFSE-DEX vs controls: mean rank difference, −1.35; *p* = 0.99). ***D***, There was no notable difference between the mean durations of the spike series among groups. *p < 0.05.

We assessed the presence, frequency, and duration of spike series during the days and weeks following eFSE in rats treated *post hoc* with DEX or vehicle (Fig. 2*A*). Neither CTL-VEH nor CTL-DEX rats developed spike series throughout the recording period, and the two groups were combined for further analysis. Representative bilateral hippocampal EEG traces from a control rat are shown in Figure 2*B*. Spike series were detected in EEGs from eFSE-VEH rats and were mainly confined to a single hippocampus (Fig. 2*C*, middle trace); a single eFSE-VEH rat had a single electrographic seizure (Fig. 2*C*, bottom trace). Notably, rats were immobile during recorded spike series, as well as during the seizure. Quantitative analyses revealed significant differences between the control and eFSE-VEH groups in the proportion of rats that had spike series (Barnard’s exact test, two-tailed, *p* = 0.0062; Fig. 3*A*). Specifically, among eFSE-VEH rats, 41.6% (5 of 12) had at least one spike series during the month following eFSE. DEX significantly reduced this proportion: only 1 of 13 eFSE-DEX rats (7.6%) had at least one spike series during the same recording time, a significantly lower risk than the eFSE-VEH group (Barnard’s exact test, two-tailed, *p* = 0.031). Indeed, the probability of having spike series was not significantly different in the control and eFSE-DEX groups (Barnard’s exact test, two-tailed, *p* = 0.37).

The number of spike series per animal was higher in the eFSE-VEH group (4.33 ± 9.96) than in controls (0 ± 0.0; Kruskall-Wallis [K-W] ANOVA for nonparametrically distributed data). ANOVA demonstrated a mean rank difference of −8.083 (*p* = 0.01, Dunn’s multiple-comparisons test; Fig. 3*B*). Importantly, the mean number of spike series per animal in the eFSE-DEX group did not differ significantly from the value in controls (K-W ANOVA: eFSE-DEX mean, 0.23 ± 0.83; mean rank difference, −1.53; *p* = 0.999). In addition, there was a >90% reduction in the mean number of spike series when the eFSE-VEH and eFSE-DEX groups were compared (eFSE-VEH, 4.33; eFSE-DEX, 0.23; mean rank difference, 6.55; *p* = 0.067).

To account for variances in recording times, we compared across groups the frequency of spike series per hour recorded and identified significant group differences (Fig. 3*C*). Spike-series frequency was higher in the eFSE-VEH group compared with controls (CTL mean, 0 ± 0, eFSE-VEH mean, 0.029 ± 0.06; K-W ANOVA: K-W statistic, 9.71; mean rank difference, −8.29; *p* = 0.01; Dunn’s multiple-comparisons test). DEX significantly reduced the frequency of spikes (eFSE-DEX vs eFSE-VEH groups, *p* = 0.046; K-W ANOVA, mean rank difference, 6.95). Indeed, spike series frequency in the eFSE-DEX group did not differ significantly from that in controls (eFSE-DEX mean, 0.0007 ± 0.002; K-W ANOVA: mean rank difference, −1.35; *p* = 0.99). The duration of each spike series was variable both within individual rats and across rats, ranging from 3 to 258 s. Because only one rat in the eFSE-DEX group had spike series, differences in the mean duration of spike series among groups could not be statistically compared. As is apparent from Figure 3*D*, the average duration of spike series in the single eFSE-DEX rat that had them was not notably different from the duration of spike series in the eFSE-VEH group.

In both humans and rodents, epilepsy develops in only a subgroup of individuals after FSE (or eFSE; Annegers et al., 1987; Dubé et al., 2006, 2010b; Choy et al., 2014). Seeking to identify predictors of epilepsy development in individual rats, we have previously identified epilepsy-predictive MRI T_2_ signal changes in limbic structures in 42% (8 of 19) of all eFSE rats at 2 h following eFSE (Choy et al., 2014). Considering this prior knowledge, we examined whether the prevalence of hyperexcitability (spike series) in the eFSE-VEH group was similar to the prior findings, and whether it was influenced by DEX administration. We used observed versus expected goodness-of-fit analysis and set the predicted/expected hyperexcitability prevalence as 45%. In the eFSE-VEH group, the observed prevalence of hyperexcitability, defined as the presence of spike series, was not significantly different from the expected 45% (*p* > 0.99). In contrast, observed prevalence in the eFSE-DEX group was significantly lower from expected prevalence (*p* = 0.009). Together, the data and analyses above demonstrated that transient administration of DEX following eFSE reduced the probability of developing a hyperexcitable hippocampal network.

### Neuroinflammatory cytokine production following eFSE is attenuated by DEX

Having identified that DEX suppressed the development of spike series after eFSE, we next addressed the potential mechanisms. Specifically, we examined whether eFSE generated an inflammatory response in the hippocampus in a subset of eFSE rats, and whether DEX attenuated this inflammation. We assessed neuroinflammation using two independent quantitative approaches: we measured the rapid upregulation of proinflammatory mRNA transcripts as well as the proliferation of astrocytes and microglia.

**Figure 4.**
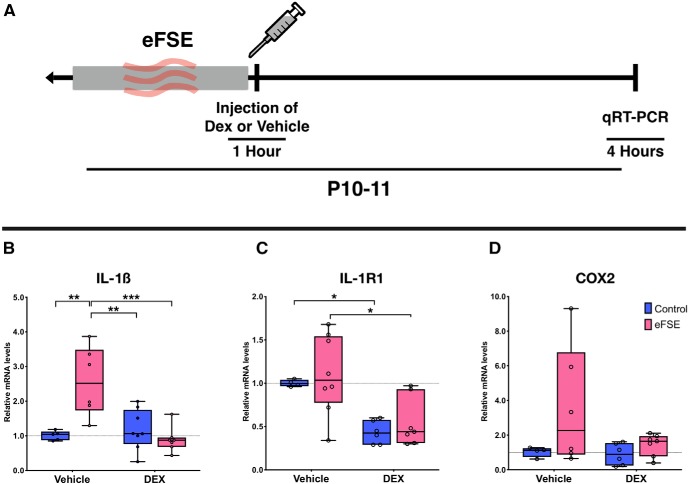
DEX administration reduces the expression of inflammatory molecules provoked by eFSE. ***A***, Experimental design: rats of both sexes underwent eFSE at P10–P11. The rats were killed and hippocampi were rapidly dissected 4 h from eFSE onset. ***B–D***, At the group level, *post hoc* eFSE increased expression of IL-1β (***B***), and IL-1R1 (***C***), DEX administration significantly decreased the mRNA expression of IL-1β and tended to reduce COX2 expression (***D***), all analyzed using two-way ANOVA followed by Tukey’s multiple comparisons. IL-1β: eFSE: 18.5% variance; *F*_(1,22)_ = 9.61; *p* = 0.005; DEX treatment: 13.3% total variance; *F*_(1,22)_ = 6.92; *p* = 0.02; IL-1R1: eFSE: 44% total variance; *F*_(1,21)_ = 18.81; *p* = 0.0003; DEX treatment: 1.6% total variance; *F*_(1,21)_ = 0.70; *p* = 0.41. eFSE-VEH versus eFSE-DEX, *p* = 0.012. COX2: effect of eFSE: 7.74% total variance; *F*_(1,19)_ = 2.09; *p* = 0.17; effect of DEX treatment: 14.39% variance; *F*_(1,19)_ = 3.88; *p* = 0.06. CTL-VEH versus eFSE-DEX, *p* = 0.98; eFSE-VEH versus eFSE-DEX, *p* = 0.20. *p < 0.05, **p < 0.01, ***p < 0.0005.

We used qRT-PCR to analyze mRNA levels of several inflammatory mediators that are rapidly augmented by eFSE (Dubé et al., 2005; Patterson et al., 2015), as well as other models of status epilepticus (Jiang et al., 2010; Vezzani et al., 2011b; Fig. 4*A*), and that have been implicated in the epileptogenesis that follows eFSE (Dubé et al., 2010a; Choy et al., 2014; Patterson et al., 2015). Levels of IL-1β mRNA in hippocampi of experimental rats 3 h after the end of eFSE were compared with those of controls. eFSE and DEX treatment both had a significant effect on IL-1β mRNA levels (two-way ANOVA: eFSE: *F*_(1,22)_ = 9.61; *p* = 0.005; DEX treatment: *F* = 6.92, *p* = 0.02) and the effects of eFSE and DEX treatment interacted (*F* = 14.36; *p* = 0.001). Thus, eFSE significantly increased the production of IL-1β in eFSE-VEH rats compared with the CTL-VEH group (*p* = 0.0018, Tukey’s multiple comparisons). There was a significant reduction in IL-1β levels in eFSE-DEX-treated rats versus eFSE-VEH treated rats (*p* = 0.0003). In fact, DEX administration after eFSE rendered the levels of hippocampal IL-1β mRNA statistically similar to those of CTL-VEH rats (*p* = 0.98; Fig. 4*B*).

Levels of IL-1R1 mRNA were also influenced by group. eFSE affected mRNA production of IL-1R1 (two-way ANOVA Tukey’s multiple-comparisons test:, eFSE: 44% of total variance; *F*_(1,21)_ = 18.81; *p* = 0.0003). DEX reduced IL-1R1 mRNA levels in eFSE-DEX rats compared with the eFSE-VEH rats (*p* = 0.012) and rendered the levels of hippocampal IL-1R1 mRNA in eFSE-DEX rats statistically indistinguishable from those of CTL-VEH rats (*p* = 0.12; Fig. 4*C*). The anti-inflammatory effect of DEX was also apparent in controls as a significant reduction in IL-1R1 levels in the CTL-DEX rats compared with the CTL-VEH rats (*p* = 0.046). mRNA levels of COX2 did not reveal statistically significant group differences (two-way ANOVA; effect of eFSE: *F* = 2.09; *p* = 0.17; effect of DEX: *F* = 3.88; *p* = 0.06). However, there was much heterogeneity in the eFSE-VEH group with regard to the expression of this proinflammatory enzyme, likely reflecting the fact that COX2 levels peak later, at 24 h after eFSE (Patterson et al., 2015). DEX administration decreased COX2 levels following eFSE so that the eFSE-DEX group was indistinguishable from the CTL-VEH group (*p* = 0.98; Fig. 4*D*).

Together, these findings suggest a rapid upregulation of proinflammatory cytokines and receptors within 3 h after eFSE. Whereas at this the early time point we did not examine protein expression, our previous studies have shown excellent congruence between inflammatory mRNA changes and corresponding variations in protein expression as measured by Western blot (Patterson et al., 2015).

### Hippocampal microglia and astrocytes: effects of eFSE and of DEX

**Figure 5.**
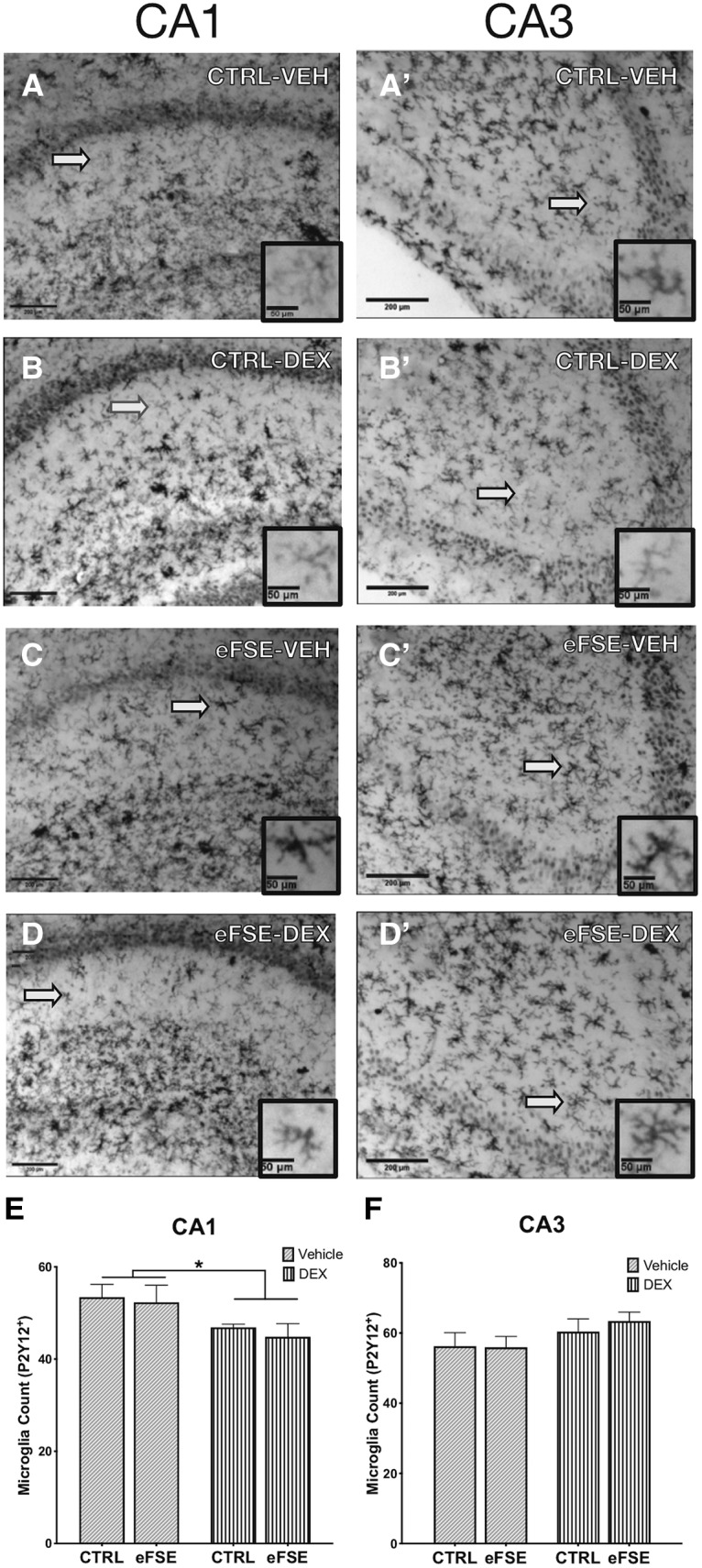
DEX reduces microglial density in both eFSE and control hippocampi. ***A–D*′**, Representative samples of P2Y12^+^ cells in the CA1 and CA3 regions of the hippocampi of CTL-VEH (***A***, ***A*′**), CTL-DEX (***B***, ***B*′**), eFSE-VEH (***C***, ***C*′**), and eFSE-DEX rats (***D***, ***D*′**). ***E***, ***F***, We did not find an increase in the number of microglia in the CA1 (***E***) or CA3 (***F***) of rats 24 h after undergoing eFSE, but DEX administration did reduce the number of microglia in eFSE and control rats (two-way ANOVA; main effect of DEX, *p* < 0.0165). Scale bars: 200 μm; inset, 50 μm. *p < 0.05.

**Figure 6.**
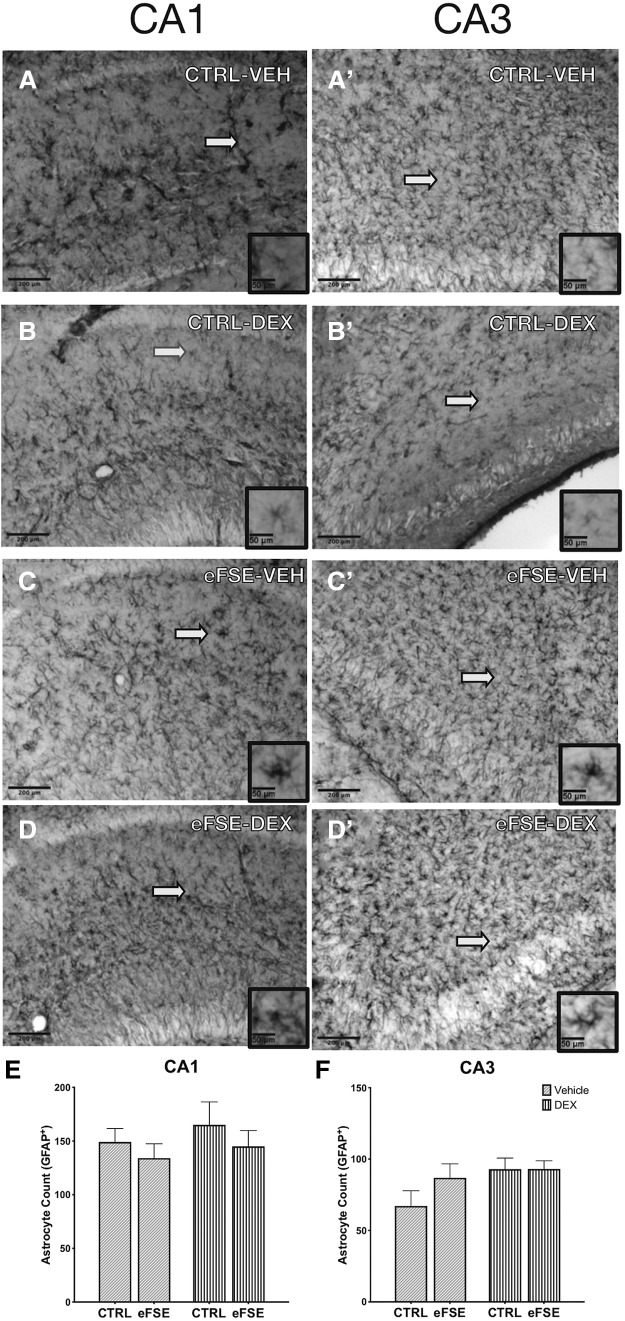
eFSE and DEX do not change the density of astrocytes following eFSE. ***A–D*′**, Representative samples of GFAP^+^ cells in the CA1 and CA3 regions of the hippocampi of CTL-VEH (***A***, ***A*′**), CTL-DEX (***B***, ***B*′**), eFSE-VEH (***C***, ***C*′**), and eFSE-DEX rats (***D***, ***D*′**). We did not find an increase in the number of astrocytes in the CA1 (***E***) or CA3 (***F***) of rats 24 h after undergoing eFSE or with DEX administration (two-way ANOVA; main effect of eFSE, *p* = 0.30; DEX *p* = 0.43). Scale bars: 200 μm; inset, 50 μm.

Proliferation and morphologic changes in the shape of microglia and astrocytes is a common inflammatory response to insults that provoke epilepsy (Aronica and Crino, 2011; Vezzani et al., 2011b). eFSE, which does not provoke cell death (Dubé et al., 2007, 2010b), results in a modest glial response. As found previously (Patterson et al., 2015), microglial density in hippocampal subfields CA1 and CA3 was not influenced significantly by eFSE (Fig. 5). Notably, in area CA1, DEX reduced microglial density in both eFSE and control hippocampi consistent with its established anti-inflammatory actions (two-way ANOVA; main effect of DEX, *p* < 0.0165). For astrocytes, our analyses at 24 h after the end of the eFSE did not reveal the effects of either eFSE or DEX (Fig. 6). Together, these findings suggest that eFSE provokes the extremely rapid mRNA expression of proinflammatory cytokines such as IL-1β, and these precede and perhaps contribute to glial proliferative reactions.

### BBB is disrupted by eFSE, an effect attenuated by DEX administration

**Figure 7.**
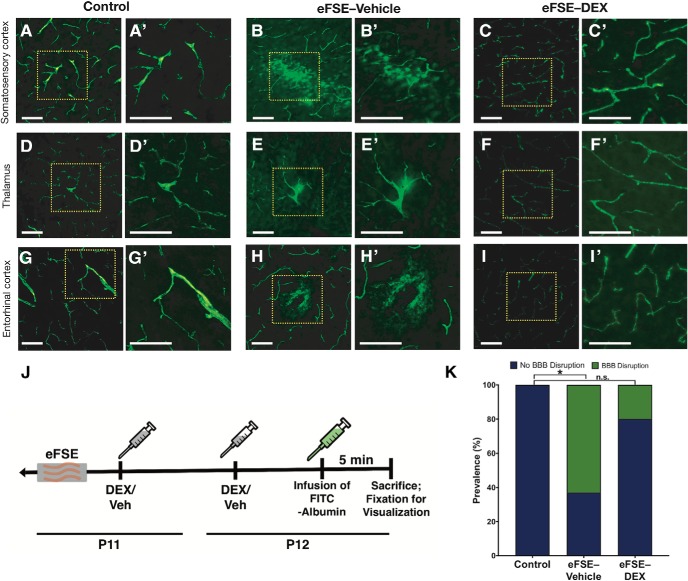
eFSE induces increased permeability of the BBB to albumin, which is attenuated by post-eFSE DEX administration. ***A–I***, Representative examples of FITC-albumin in control rats, eFSE-VEH rats, and eFSE-DEX rats in the somatosensory cortex, thalamus, and entorhinal cortex. Extravasation can be seen in ***B***, ***E***, and ***H*** as the FITC-albumin spreading outside of the vasculature. ***J***, Experimental design: rats underwent eFSE at P11 and received two tapering doses of DEX. Rats were infused with FITC-albumin and killed 5 min later. ***K***, There was a significant increase in prevalence of rats that had extravasation between controls versus eFSE-VEH (Fig. 6*K*; Barnard’s exact test, two-sided, *p* = 0.001; score, −3.162), but no difference between controls and eFSE-DEX, revealing that DEX treatment following eFSE reduces the risk of BBB extravasation to that of control rats (Barnard’s exact test, two-sided, *p* = 0.12; score, −1.60. *p < 0.05. n.s., Not significant. Scale bar, 100 μm.

We examined whether augmented permeability of the BBB takes place after eFSE and may contribute to the observed hippocampal neuroinflammatory cascades that promote epileptogenesis (Bar-Klein et al., 2017; Kim et al., 2017; Koepp et al., 2017; Rüber et al., 2018). To visualize BBB permeability, we used an infusion of FITC-albumin, which is typically excluded from the brain by the BBB tight junctions (Fig. 7*J*; Wolburg and Lippoldt, 2002). Sections from brains of control rats, regardless of DEX administration (*n* = 12), did not reveal any extravasation of the FITC-albumin (Fig. 7*A*,*A*′,*D*,*D*′,*G*,*G*′). Therefore, the DEX- and vehicle-receiving control groups were combined for further analyses. In contrast, abnormal extravasation of FITC-albumin was apparent in sections from five of eight eFSE-VEH rats (62.5%), measured as increased fluorescence within the parenchyma and occasionally within somata (Fig. 7*B*,*B*′,*E*,*E*′,*H*,*H*′). Common areas of BBB breach included cortex, both somatosensory and entorhinal (Fig. 7), and the mediodorsal nucleus of the thalamus. Notably, extravasation was not observed in hippocampus. Statistical analysis identified a significant difference between controls versus eFSE-VEH (Fig. 7*K*; Barnard’s exact test, two-sided, *p* = 0.001; score, −3.162), but no difference between controls and eFSE-DEX, revealing that DEX treatment following eFSE reduces the risk of BBB extravasation to that of control rats (Barnard’s exact test, two-sided, *p* = 0.12; score, −1.60).

## Discussion

Here we find that (1) eFSE generates a proepileptogenic hyperexcitable hippocampal network; (2) this hyperexcitability, apparent as the presence of spike series, is accompanied by an acute molecular and cellular neuroinflammatory reaction and a breach of the BBB; (3) these disturbances occur in a subset of rats, in accord with FSE-provoked epileptogenesis in rodents and humans; and (4) the proepileptogenic consequences of eFSE are attenuated by a *post hoc* short course of the clinically available drug dexamethasone.

### Spike series are a surrogate marker of eFSE-induced epileptogenesis and enable designing mechanistic interventional studies

In children, TLE develops in a subgroup of those experiencing FSE with 10–12 year latency (Seinfeld et al., 2016), which prohibits the design of adequately powered intervention strategies. (Hesdorffer et al., 2012). Therefore, as is common throughout clinical intervention trials, markers are sought that can be detected early and indicate a high risk for eventual disease (Barker-Haliski et al., 2014). For FSE, the FEBSTAT study obtained early EEGs, cytokines, and MRIs as potential surrogate markers of epileptogenesis (Nordli et al., 2012; Lewis et al., 2014; Gallentine et al., 2017). In the current animal model, only 40% of eFSE rats develop spontaneous seizures, and these are sparse (Choy et al., 2014). To avoid the prohibitive cost of adequately powered studies (required, *n* = 199/group; see Materials and Methods) required to demonstrate a significant reduction in seizure prevalence or frequency from already low levels, we used a surrogate marker. We established that spontaneous seizures never occurred without prior spike series, and, whereas not all animals with spike series developed epilepsy, spike series were a sensitive surrogate marker for epileptogenesis. Compared with the predictive MRI signal identified previously (Choy et al., 2014), spike series are a more direct indicator of aberrant neuronal firing, useful for efficient preventative trials. Here we examined the effects of DEX on this surrogate marker (Barker-Haliski et al., 2014).

### Acute rapid inflammatory processes initiated by eFSE in a subset of individuals require early intervention

Neuroinflammation has been demonstrated after human FSE and in rodent FSE models (Heida and Pittman, 2005; Reid et al., 2013; Gallentine et al., 2017). We have previously found that inflammatory cytokines are both required for eFSE generation and are upregulated by the seizures (Dubé et al., 2005, 2010b; Patterson et al., 2015). Furthermore, whereas only a subset of rats sustained inflammation, these same rats had MRI changes that predicted epileptogenesis (Patterson et al., 2015). Here we demonstrate rapid elevations of IL-1β, IL-1R1, and COX2 already at 3 h from the eFSE. While this elevation was not significant on group levels for IL1-R1 and COX2, it occurred in ∼40% of rats, which is consistent with previous work and the proportion of rats developing epilepsy (Patterson et al., 2015). The remarkable rapidity of the inflammatory response that follows eFSE provides the rationale for initiating anti-inflammatory intervention within hours of the eFSE, as done here.

### eFSE-induced neuroinflammation: a function of age and seizure type?

We further delineated the sequence of neuroinflammatory events initiated by eFSE and lasting for a minimum ∼72 h (Dubé et al., 2010b; Patterson et al., 2015). Prior work identified translocation of high-mobility group box1 (HMGB1) from neuronal nuclei to cytoplasm for export, within an hour from eFSE (Choy et al., 2014). HMGB1 interacts with glial inflammatory receptors (e.g., toll-like receptor 4), activating astrocytes and microglia. Here, cytokine mRNA production was found already within 3 h from the end of the eFSE, and protein expression was confirmed previously using Western blots. At single-cell resolution, immunohistochemistry showed IL1β production in astrocytes but not in microglia (Dubé et al., 2010b). IL-1β production does not derive from the proliferation or migration of astrocytes and microglia: even by 24 h after eFSE, there was no hippocampal astrocytosis or microgliosis. The increased levels of IL-1β production, observed already at 3 h, may contribute to BBB disruption, which was apparent by 24 h after eFSE (Vezzani and Baram, 2007).

The neuronal to glial progression of neuroinflammation and its distinct molecular patterns may stem from the fact that eFSE takes place in an immature brain and does not provoke cell death, likely because of the resilience of immature neurons to excitotoxicity (Dubé et al., 2000; Sullivan et al., 2003). Hence, the inflammation is not a response to neurodegeneration and may contribute directly to the formation of an epileptic network (Vezzani and Baram, 2007).

### Disruption of the BBB 24 h after eFSE

The current work uncovered BBB disruption by eFSE. Whereas the BBB is not fully mature in neonatal rats, it appears to be functional in P12 Sprague Dawley rats reared in defined conditions that standardize body weight and brain development. Thus, in control pups, the BBB excluded FITC-albumin from brain parenchyma in 12 of 12 pups. In contrast, BBB permeability was increased in a subset of eFSE rats 24 h later, with FITC-albumin extravasation in the entorhinal, frontoparietal, and piriform cortices, and the mediodorsal nucleus of the thalamus. These areas of disruption are intriguing for two reasons. First, they are a component of the limbic circuitry involved in TLE-like limbic epilepsy. In addition, prior literature on SE-induced injury in the developing brain has identified the medial thalamus as sensitive to injury after SE in immature rats (Kubová et al., 2002), and MRI changes in medial thalamus were predictive of epileptogenesis after eFSE (Choy et al., 2014). Additionally, the piriform cortex has been shown to be particularly sensitive to SE-provoked BBB breakdown, and this permeability was predicative of epileptogenesis (van Vliet et al., 2014; Bar-Klein et al., 2017). Interestingly, BBB disruption was not observed in the hippocampus per se, but rather in the interconnected cortical and thalamic regions noted above.

The role of BBB in epileptogenesis has been a topic of intense study. Support for the mechanistic role of BBB disruption in epileptogenesis was provided by Bar-Klein et al., 2014, who demonstrated antiepileptogenic effects of targeting the processes underlying BBB disruption. We can only speculate if BBB disruption after eFSE is causally involved in epileptogenesis. At a minimum, the BBB leakage represents a component of an inflammatory cascade that was attenuated by early administration of DEX.

### The rationale for DEX, and its promise

The combined findings of this and prior studies highlight the fact that multiple inflammatory cascades in several types of cell pathways are initiated by eFSE. These pathways are intercalated via numerous feedback loops and compensatory processes. This suggested that interfering specifically with only one of these processes might not suffice to prevent epileptogenesis, prompting the use of the broad anti-inflammatory agent DEX.

Indeed, a number of more specific other anti-inflammatory approaches have not been successful in preventing epileptogenesis. Holtman et al. (2009, 2010) used several agents including COX2 inhibitors with little success in adult SE. Jiang et al. (2010, 2013) found neuroprotective, but not antiepileptogenic, effects of blocking the EP2 pathway. These studies and others have suggested that selective approaches to specific inflammatory cascades might have limited potential, at least in part because of the complex interplay and balance among the numerous inflammatory processes triggered by insults, a view supported by Brennan et al. (2016), who targeted miR124. Whereas, more recently, Terrone et al. (2018) reported on success in attenuating epileptogenesis by targeting the IL-1β pathway and Henshall (2017) capitalized on the ability to manipulate microRNAs in adults, we chose here to use a broad anti-inflammatory approach, hoping to obviate feedback or compensatory changes within the complex inflammatory network (Vezzani et al., 2011a; Brennan et al., 2016).

DEX is a broad and powerful anti-inflammatory agent, approximately five times more potent than prednisone, and, at the doses used here, it rapidly crosses the blood–brain barrier, a concern whenever dealing with therapeutics in the brain (van de Waterbeemd et al., 1998). Additionally, DEX is clinically available and widely used in children; its costs and mode of administration are manageable, enabling potential clinical application. Therefore, the finding that DEX significantly attenuated both proinflammatory mediators and spike series, a measure of network hyperexcitability, is encouraging.

### Potential limitations and future studies

The aberrant hyperexcitability addressed in the current studies was elicited by eFSE in immature rats. TLE associated with eFSE involves minimal cell death and might thus be easier to prevent than chemoconvulsant-induced epileptogenesis in adults. In contrast, the nature and duration of the insult, the age of affected individuals, MRI changes, and the proportion of individuals who develop spontaneous seizures approximate those in humans experiencing FSE. Therefore, our results, while not generalizable to adults, should carry major significance for FSE-related TLE.

Several questions remain. Memory and related cognitive problems develop in children experiencing FSE (Weiss et al., 2017); we have recently identified these problems in eFSE rats (Dubé et al., 2009; Patterson et al., 2017). Future work will test whether DEX has protective effects on cognition. Notably, we used a high DEX dose. While no obvious deleterious effect was noted in acutely DEX-treated rats, dosing and potential adverse effects should be addressed.

In summary, the contribution of FSE to common and often serious epilepsy has been established. Here we provide novel evidence for the role of brain inflammation in the epileptogenesis that follows eFSE. Importantly, we attenuated eFSE-induced hippocampal hyperexcitability by dampening inflammatory mediators using a clinically available anti-inflammatory agent.

## Synthesis

Reviewing Editor: Quentin Pittman, University of Calgary Decisions are customarily a result of the Reviewing Editor and the peer reviewers coming together and discussing their recommendations until a consensus is reached. When revisions are invited, a fact-based synthesis statement explaining their decision and outlining what is needed to prepare a revision will be listed below. The following reviewer(s) agreed to reveal their identity: NONE. Note: If this manuscript was transferred from JNeurosci and a decision was made to accept the manuscript without peer review, a brief statement to this effect will instead be what is listed below.

The editor believes that the authors have persuasively made their point concerning the major issues raised by the reviewer #3. It would be impractical to attempt to carry out th elong term recordings after eFS demanded by the reviewer. I agree that the EEG recordins from neonatal animals are not pretty, but these are difficult to obtain and one can see much better ( or more expected) ones in the adult animals.

Finally, as noted by the reviewer the relationship to BBB breakdown is indeed correlational and not necessarily causal, and the authors do possilby attempt to make too much of this; however, this is an interpretative matter that can be left to the discretion of the reader
